# Leading Learning Health Systems: Strategies Used to Establish and Conduct a Learning Health System

**DOI:** 10.1007/s11606-025-09849-0

**Published:** 2025-09-25

**Authors:** Dawn M. Bravata, Cathy C. Schubert, Amy M. Kilbourne, Scotte R. Hartronft, Ashley L. Schwartzkopf, Jennifer L. Myers, Mindy E. Flanagan, Claire E. Donnelly, Stanley E. Taylor, Mathew J. Reeves, Anthony J. Perkins, Laura E. Myers, Indrakshi Roy, Robert E. Burke, Rachael A. Beard, Marcos Montagnini, Shivani K. Jindal, Greg Arling, Caroline Madrigal, Nicholas A. Rattray

**Affiliations:** 1 Department of Veterans Affairs (VA) Health Systems Research (HSR) Expanding Expertise Through E-Health Network Development (EXTEND) Quality Enhancement Research Initiative (QUERI), Indianapolis, IN USA; 2 VA HSR Center for Health Information and Communication (CHIC), Richard L. Roudebush VA Medical Center, Indianapolis, IN USA; 3 Division of General Internal Medicine and Geriatrics, Department of Medicine, Indiana University School of Medicine, Indianapolis, IN USA; 4 Regenstrief Institute, Indianapolis, IN USA; 5 Community, Home, and Geriatrics Service, Richard L. Roudebush VA Medical Center, Indianapolis, IN USA; 6 Office of Research and Development, Veterans Health Administration, U.S. Department of Veterans Affairs, Washington, DC USA; 7 Department of Learning Health Sciences, University of Michigan, Ann Arbor, MI USA; 8 Office of Geriatrics & Extended Care, Veterans Health Administration, U.S. Department of Veterans Affairs, Washington, DC USA; 9 Department of Epidemiology and Biostatistics, Michigan State University, East Lansing, MI USA; 10 Department of Biostatistics and Health Data Science, Indiana University School of Medicine, Indianapolis, IN USA; 11 Department of Epidemiology and Biostatistics, Indiana University School of Public Health, Bloomington, IN USA; 12 VA HSR Center for Healthcare Evaluation, Research, and Promotion (CHERP), Crescenz VA Medical Center, Philadelphia, PA USA; 13 Perelman School of Medicine, University of Pennsylvania, Philadelphia, PA USA; 14 Office of Nursing Services, Veterans Health Administration, U.S. Department of Veterans Affairs, Washington, DC USA; 15 Nursing Service, St. Louis VA Healthcare System, St. Louis, MO USA; 16 VA Geriatric Research Education and Clinical Center (GRECC), Ann Arbor, MI USA; 17 Veterans Integrated Service Network (VISN 10) Geriatrics, Ann Arbor, MI USA; 18 Division of Geriatric and Palliative Medicine, Department of Internal Medicine, University of Michigan, Ann Arbor, MI USA; 19 Medical Service, Cincinnati VA Medical Center, Cincinnati, OH USA; 20 Department of Internal Medicine, University of Cincinnati College of Medicine, Cincinnati, OH USA; 21 School of Nursing, Purdue University, West Lafayette, IN USA; 22 VA Boston Healthcare System, Boston, MA USA

**Keywords:** Learning Health Systems, Quality improvement, Communities of practice, Implementation science, Translational science

## Abstract

**Background:**

Learning health systems (LHS) improve patient and provider experiences, population health, and health system performance. LHS leaders develop LHS’ social and scientific infrastructures and align the LHS with host organization’s priorities. Although researchers have examined data infrastructure and Learning Community configurations, few studies have evaluated discrete strategies leaders deploy to construct a LHS.

**Objectives:**

We described methods used to establish the Department of Veterans Affairs (VA) Geriatric LHS (GLHS) supporting older Veterans transitioning from hospital to home and examined strategies GLHS leaders used to conduct LHS activities.

**Design:**

Qualitative methods were employed to review notes from all meetings and post-meeting debriefs from encounters with operational partners, Learning Community members, and Quantitative and Qualitative Data Core subcommittees. Member checking with Learning Community and Data Core representatives led to iterative refinement of findings. A seven-question survey examined Learning Community member satisfaction with the GLHS.

**Results:**

Ten of 16 Learning Community members (62.5%) completed surveys, indicating that the GLHS met Learning Community members’ needs. The four key GLHS structural elements were (1) the imperative of maintaining an Idea Repository, (2) the value of notetaking and formal debriefs, (3) the cadence of meetings, and (4) the role of the newsletter to promote engagement. Five themes described core activities/perspectives GLHS operational leaders used: (1) listening to the Learning Community; (2) modifying analyses in response to Learning Community input; (3) including diverse perspectives; (4) managing organizational complexity; and (5) serving the healthcare system.

**Conclusions:**

The GLHS illustrates the importance of active listening, collaborative engagement, and responsive adaptation to advance LHS objectives. LHS leadership strategies such as an Idea Repository and iterative feedback mechanisms fostered stakeholder participation and informed data-driven improvements.

**Supplementary Information:**

The online version contains supplementary material available at https://doi.org/10.1007/s11606-025-09849-0.

## INTRODUCTION

The Learning Health Systems (LHS) model is increasingly being adopted across diverse settings to improve health system functioning and outcomes.^1,2^ Individuals responsible for implementing LHS must develop the social and scientific infrastructure within the context of the healthcare system’s strategic priorities. Although the LHS literature describes data infrastructure and Learning Community configurations supporting the LHS model, few reports describe strategies LHS leaders deploy to establish effective LHS and promote system functioning.^3,4^ There has been a call for guidance to support healthcare systems as they establish LHS.^5^

The United States Department of Veterans Affairs (VA) is the largest integrated healthcare system in the country, with a common electronic health record, robust research enterprise, and commitment to quality improvement—embodying the principles of the LHS.^6^ In 2023, the VA Health Systems Research Quality Enhancement Research Initiative (QUERI) called for proposals to establish LHS infrastructures. The rationale to develop the VA Geriatrics Learning Health System (GLHS) was based on six elements: imperative to support Veterans as they age; economic importance of aging-in-place (VA pays for nursing home care for eligible Veterans); strength of existing data infrastructure; high degree of VA geriatrics leadership engagement; high risk of adverse outcomes for older Veterans transitioning from inpatient to outpatient settings; and extensive body of evidence-based transitional practices supporting community-dwelling older persons.^7–13^

The VA GLHS was launched February 2024 to support community-dwelling older Veterans with a focus on transitional care. This study aimed to describe the GLHS and examine strategies GLHS leaders used to conduct GLHS activities; we sought to describe approaches that teams establishing LHS could use to implement the LHS model.

## METHODS

We considered conceptual frameworks to guide data collection and analysis: LHS maturity grid assessment,^14^ Kaiser Permanente Washington Logic Model,^15^ and framework for value-creating Learning Health Systems.^16^ Although these models describe aspects of LHS leadership (e.g., participatory leadership^16^), they did not provide a structure for evaluating LHS leadership activities. In alignment with Friedman’s LHS conceptualization^17^ and the application of LHS to inform health policy,^18^ we embraced the concept of co-creation^19^ within a LHS guided by a Learning Community (LC) (a multistakeholder group motivated to address a healthcare problem that directs LHS learning cycles of discovery to implementation^17^). Therefore, we used an inductive approach focused on emergent themes^20^ to describe structures, strategies, and perspectives used by GLHS leaders. This non-research work was conducted as quality improvement.

### Geriatric Learning Health System

**Figure 1:**
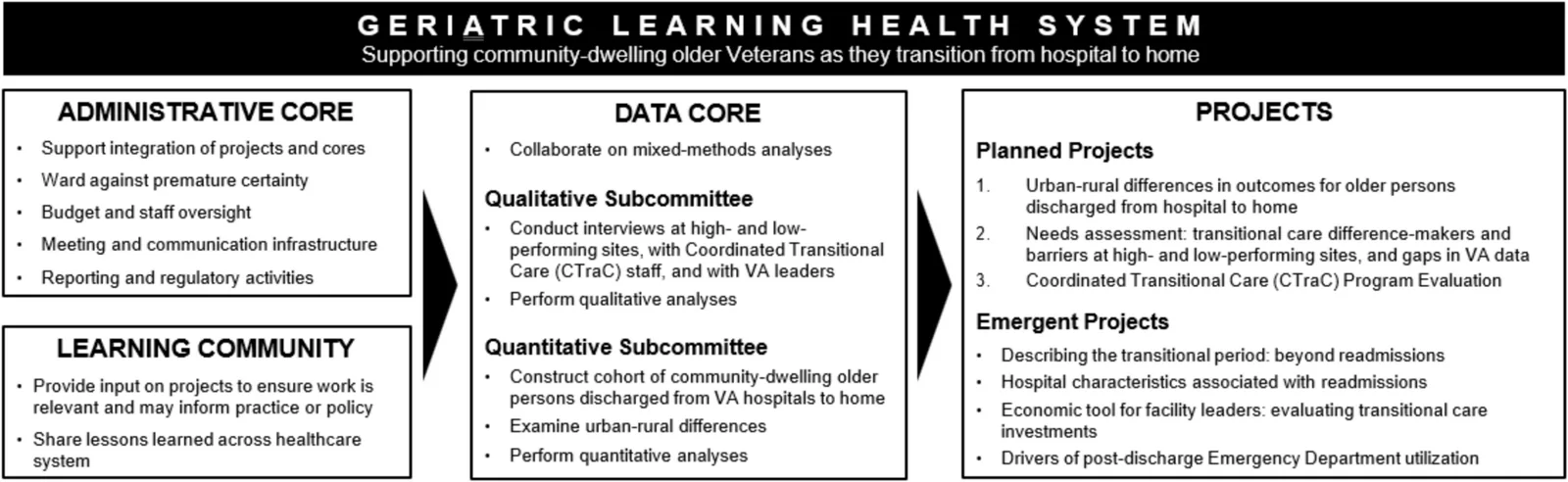
The Geriatric Learning Health System: projects and cores. Describes the Geriatric Learning Health System which is focused on supporting community-dwelling older Veterans as they transition from hospital to home; specifically, three cores support the portfolio of work which includes both main projects and emergent projects.

The GLHS was funded as an 18-month infrastructure project to establish three cores (LC, Data Core, Administrative Core) and conduct three projects (Fig. 1).

We assembled and facilitated a Learning Community (LC) of 16 operational leaders with policy-making authority positioned to make decisions based on lessons learned from the GLHS; leaders were from geriatrics, hospital medicine, nursing, primary care, social work, and rehabilitation. Several LC members had academic affiliations. Members of the LC lived across the country; therefore, all LC meetings were conducted virtually via Microsoft Teams. To begin building a sense of community, we first held a 1-day kickoff including introductions and ice breaker activities in addition to discussion of project objectives. Thereafter, LC meetings were convened at least monthly to maintain engagement and project momentum. The leadership team circulated agendas ahead of time to allow LC members to prepare and remind them about upcoming meetings. Additionally, the leadership team reached out to individual LC members before meetings if topics were in their areas of expertise, to highlight the importance of their participation. LC meetings focused on a variety of topics: project design (e.g., metrics to evaluate transitional care, approach to identifying high- and low-performing sites for interviews, key processes of care to examine), project results, transitional care program innovations, brainstorming sessions about work needed to inform practice or policy, and conversations about how VA leaders learn about innovations and disseminate best practices. Every meeting began with a reminder about the inclusive, collaborative culture of listening and learning we sought to establish. LC members were encouraged to participate with cameras on to foster the sense of community and were called on during meetings to encourage full participation. Sessions involving prioritization of ideas used the Poll Everywhere voting application. Informal “office hour” sessions held approximately every other month accommodated LC members unable to attend the formal monthly session.

The Data Core (*N* = 19) had two subcommittees. The Qualitative Subcommittee had 7 qualitative methodologists and implementation scientists from diverse backgrounds: anthropology, psychology, primary care, nursing, and social work. The Quantitative Subcommittee had 11 methodologists with expertise in data science, clinical epidemiology, biostatistics, sociology, economics, and health services research.

The Administrative Core included five individuals with clearly defined responsibilities: two co-directors (a geriatrician [CCS] with service-line operations and program implementation experience led the LC, and a general internist [DMB] with experience in partnered research, national VA observational cohorts, and quality improvement led the Data Core Quantitative Subcommittee); two co-administrators with clinical social work backgrounds (JLM oversaw human resources, budgets, and managed the Data Core Qualitative Subcommittee; ALS oversaw communication across units, established meeting schedules, and managed the Data Core Quantitative Subcommittee including data acquisition); and a medical anthropologist and implementation scientist (NAR) led the Data Core Qualitative Subcommittee. All five were senior VA staff with existing relationships with operational partners and each other, and established reputations within the VA community.

The three projects were as follows: a needs assessment to identify clinical practices associated with transitional success at high-performing sites, transitional care needs at low-performing sites, and data infrastructure or analytic needs; an assessment of urban–rural differences in transitional outcomes; and a mixed methods assessment of the Coordinated Transitional Care (CTraC) program.^13,21,22^

### Data Collection and Analysis

Members of the three cores participated in a range of meetings. At each meeting, observational notes were collected by at least one team member using a template developed for rapid analysis.^23^ The template included a brief overview of each session, prompts about interactions and points of interest, and reflections to guide team discussion and adaptations for future meetings (Supplement A). Most meetings were video-based, which were recorded and auto-transcribed. Following monthly LC meetings, the GLHS leaders held 30-min debriefing sessions to reflect on ongoing implementation and plan future modifications.

We comprehensively reviewed notes and post-meeting debriefs from meetings with operational partners (VA leaders across disciplines, and from varying levels within the healthcare system, who were not members of the LC), the LC, and Data Core, and composed analytic memos.^24^ We used member checking^25,26^ to ensure findings represented experiences of the Administrative Core. We used critical participatory looping^27^ to generate co-constructed conversations about preliminary results with LC (RAB, REB, SRH, SKJ, MM) and Data Core representatives (CM, AJP, MR, SET). We iteratively revised results based on two video conferences and review of written findings. During member checking, we asked team members to consider whether preliminary findings aligned with their personal experience participating in GLHS activities (did the results “feel right”); if key themes or activities were missing; and which activity, structure, strategy, or perspective was most salient or helpful (most important to share with people launching a LHS).

We also developed and fielded a seven-question, anonymous, online (Research Electronic Data Capture [REDCap], Nashville, TN) survey (Supplement B) with LC members to evaluate short-term GLHS effectiveness. Existing learning organization surveys were reviewed to understand relevant constructs for evaluating the GLHS.^28–30^ The Data Core Qualitative Subcommittee considered existing surveys and goals for the early-stage GLHS, and developed a survey to assess effectiveness of meeting and activities, satisfaction, and learning.

## RESULTS

The following three sections provide results describing: GLHS structures, the LC survey, and thematic analysis of GLHS leadership approaches.

### GLHS Structural Elements

**Figure 2:**
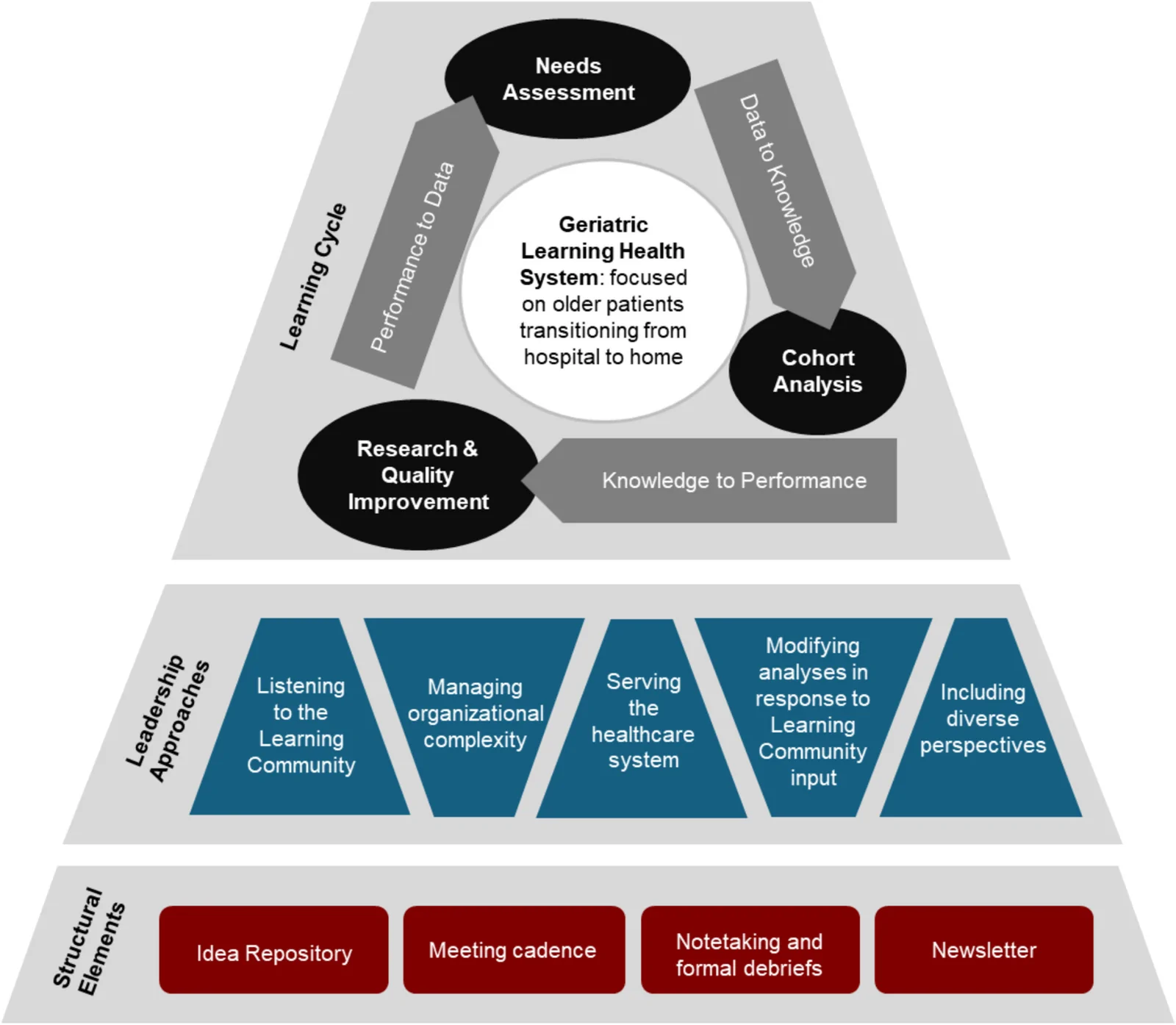
Structural elements and leadership approaches supporting learning cycles within the Geriatric Learning Health System. Displays how the structural elements and leadership approaches support learning cycles within the Learning Health System model

Four GLHS structural elements are highlighted (Table 1; Fig. 2): imperative of maintaining an Idea Repository; value of notetaking and formal debriefs; meeting cadence; and role of the newsletter to promote engagement.

**Table 1:** Structural Components of the Geriatric Learning Health System

Structure or strategy	Description*	Function
Formal meetings	• Kickoff: 3.5 h meeting to launch GLHS • LC (LC): 1 h, monthly • Combined Data Core (DC): 1 h, monthly • Data Core subcommittees: each are 1 h, weekly • Administrative: 1 h, weekly	• Reminding LC and DC of our status within 18-month timeline • Administrative meetings to review milestones and identify barriers to progress • Saying thank you, welcoming individuals, using preferred names during meetings and post-meeting follow-up • Often involved presenting preliminary project data, information about VA programs, or facility characteristics to the LC for them to respond to within group brainstorming sessions to generate guidance for project development and analyses
Office hours	• Monthly informal discussions	• Support ongoing conversations centered on the same topic as that month’s LC meeting
Ad hoc meetings	• Videoconferences: 6–15 per month, each 30–60 min	• To meet with individuals who have been identified as potential members of the LC or DC or to support the larger VA community in their work
Notetaking and debriefs	• Formal notetakers for LC • Formal debriefs: 30 min	• To ensure that all ideas and suggestions are captured • To understand interpersonal dynamics and participant engagement • To identify opportunities to improve meeting structure or content
Newsletter	• Sent out monthly embedded in email (no “clicking” required to open file) the week before LC meeting to remind about date and time • Colorful • Brief: not exceeding one page	• Biographies highlight members and foster relationship building and engagement • Update on projects serves as communication strategy across groups and opportunity to celebrate successes • Timeline tracker reminds LC and DC of where we are in terms of our 18-month timeline
Engagement tracking	• Conducted during LC meetings	• Identifies members who may benefit from outreach to enhance participation
Directory	• Includes contact information and photographs of GLHS members	• Being included in the Directory is a sign that the individual is part of the GLHS, supporting team-building and engagement
The Idea Repository	• Provides a venue for collecting ideas for analyses, program evaluations, or quality improvement that emerge during the conduct of the planned work or in response to shifting stakeholder priorities	• Allows us to acknowledge important ideas as they emerge even if we cannot accommodate them given constraints on our scope of work • Ideas could serve as topics for future LHS cycles or new programs • Periodically sharing ideas with the LC, and VA research community • A key product of the GLHS

^*^LC refers to Learning Community; DC to Data Core; and GLHS to Geriatric Learning Health System

#### Idea Repository

Learning Health Systems prioritize iterative, data-driven problem identification and improvement.^31,32^ Given that the GLHS was restricted to 18 months, we had to develop an approach to collecting ideas for work considered important by the LC but beyond the scope of our planned work. To this end, we created and maintained an Idea Repository comprised of project ideas or analyses for future LHS cycles, quality improvement, or research. The Idea Repository provided a vehicle for valuing LC suggestions and sharing ideas with operational partners, funders, and investigators. This “idea sharing” is a core LHS element but may be uncomfortable for some investigators less accustomed to disclosing intellectual property. LC members stated that the Idea Repository was a key GLHS product. We fielded requests for Idea Repository updates from VA research leaders setting priorities and diverse research teams. The rationale for an Idea Repository is grounded in three constraints: budget limitations supporting only one LHS learning cycle; therefore, emergent ideas for additional learning cycles could be added to the Idea Repository; a topic might require expertise not present within an existing LC or Data Core; and time constraints might not allow for additional analyses, development of needed expertise, or data acquisition.

#### Notetaking and Formal Debriefs

We implemented notetaking for the kickoff and LC meetings to document the content of conversations as well as impressions about body language and engagement. We convened formal debriefs with the Administrative Core and Qualitative Subcommittee (~ 30 min) to describe what went well and opportunities for improvement. Structured debriefing has been shown to support post-event learning.^33,34^ Debriefing provided a mechanism to systematically evaluate strengths and weaknesses in the conduct of LC meetings and make prospective adjustments; identify issues requiring follow-up; and identify less engaged participants who might benefit from outreach. For example, when we observed that some LC members were not able to attend sessions due to schedule conflicts, we implemented additional “office hours” and modified the meeting schedule.

#### Cadence of Meetings and Interactions

LC members reported that in contrast to other groups where participation was less frequent, monthly formal meetings and office hours provided a cadence and frequency of meetings which served to enhance engagement, foster relationship-building, provide an opportunity to respond to LC feedback in a timely fashion, and allow for learning *about* and not just *of* programs or projects (facilitating in-depth learning into potentially unfamiliar topics). LC members reported that the GLHS structure (initial kickoff followed by monthly LC meetings and office-hours) provided opportunities for members to learn from colleagues—whom they value for their expertise which differs from their own; have time to reflect on what they are learning; make decisions in the context of a broader community of stakeholders than is usually possible; and consider issues across a longer time frame than is typical. Although we sought opinions and suggestions of LC members at each meeting, we varied the meeting content (e.g., inviting outside guests to describe programs or present information, engaging participants in discussions about preliminary project results).

#### Newsletter

One approach to knowledge management and team-forming was our newsletter. The newsletter—designed using colorful blocks and not exceeding one page—was sent to LC members embedded within an email so no “clicking” was required. It was distributed the week before monthly LC meetings to remind participants about meetings. Each newsletter included a photograph and brief biography of a GLHS participant, update on projects and lessons learned, a timeline tracker functioning as a visual reminder of the status against the 18-month timeframe, and a “teaser” about emerging results to promote attendance at the monthly meeting. When asked about the newsletter, LC members particularly appreciated the biography section. Members explained that they read and shared the newsletter with constituents, highlighting its role supporting active engagement.

### LC Satisfaction Survey Results

The survey was distributed to LC members (*N* = 16); 10 responded (10/16, 62.5%); results suggested that the GLHS met LC needs. All respondents (10/10, 100%) agreed with the statement, “this LC is more appealing or useful to me than other similar professional communities in which I am a member,” offering that the reason for their satisfaction was because the GLHS offered more opportunities for learning. One respondent reported: “I love the innovative format and the discussions that happen during our LC meetings. I also enjoy collaborating with experts in the field.” LC members (9/10, 90%) reported sharing GLHS-derived information with others in the healthcare system.

### GLHS Leadership Themes

Five themes described structures, strategies, activities, or perspectives GLHS operational leaders used: listening to the LC; modifying analyses in response to LC input; including diverse perspectives; managing organizational complexity; and serving the healthcare system (Table 2; Fig. 2).

**Table 2:** Key Aspects of Leading the Geriatric Learning Health System

Theme	Perspective	Reflection*
Listening to the LC	• Valuing listening and reflecting • Valuing stakeholder time, attention, and expertise • Willing to convene many formal and ad hoc meetings to follow-up on what we are hearing • Fighting against the threat of premature certainty	• People are willing to meet with us, share their ideas and expertise • It is not always obvious the first time we hear something that it will be important. Having a core group of people who are listening is key to “hearing” messages being repeated • Processes occur over time and within the context of maturing personal relationships • Establishing a culture of collaboration and co-production requires explicit attention to fostering relationships and creating a pleasant environment for sharing ideas • Debriefs and regular administrative meetings provide a forum to reflect on what we hear and identify opportunities to improve future interactions • The cultural imperative of serving the LHS because it is advancing VHA for the care of Veterans is evident in LC, GLHS leadership, and DC
Modifying analyses in response to LC input	• Embracing the LHS model of soliciting LC input on analyses and having those ideas inform analyses • Willing to meet with people to learn about programs, data, and refine analyses	• Works best if people leading analyses are present for the LC discussion so that a direct conversation can take place with a sharing of ideas and rationale for decisions, but if that is not possible, requires leadership to communicate across groups and prioritize suggested analyses • Unlike a research project where a grant is peer-reviewed and an analysis plan guides work, the LHS iterative approach requires many ad hoc and group meetings to clarify analyses and ensure methodological rigor • Having senior methodologists who are experienced with working with operational partners facilitates this process
Including diverse perspectives	• Willing to meet with new people, identify areas of shared interest, and make them feel welcomed and valued	• LC members share their diverse opinions and would likely do so because of who they are as individuals, but we also explicitly ask them to share and seek to provide forums for discussion that value diverse perspectives (e.g., calling on people who we suspect may have alternative perspectives) • Incorporating non-VA team members requires investment of time to educate about VA (e.g., programs, data) but provides critical context for work
Managing organizational complexity	• Sharing lessons across groups is critical to the success of the LHS	• LHS must have adequate administrative leadership resources to function • It is helpful to have senior VA personnel in the administrative leadership who are familiar with VA processes and data and who already have existing relationships with stakeholders • Also helpful to have existing relationships amongst DC members • Day-to-day operations are directed by GLHS leadership with operational partners input
Serving the healthcare system	• Willing to meet with people to help them with their work • Willing to share intellectual property: ideas, data	• This work is part of system building (to advance VHA) that is neither quality improvement nor research

^*^LC refers to Learning Community; DC to Data Core; GLHS to Geriatric Learning Health System; VA to the Department of Veterans Affairs

#### Listening to the Learning Community

GLHS leaders regularly asked LC members to describe issues of importance to their strategic plans, listened and documented what LC members said, followed up on suggestions, and then fed back results of the follow-up. These activities required explicit attention to community-building and establishing a culture of collaboration. Responsive listening occurred by recognizing that LC members’ time and attention were among the most valuable resources the GLHS possessed; embodying the belief that GLHS leaders served the LC; valuing opportunities to hear from the LC; and creating meeting agendas that allowed for productive conversation. LC members reported during the member checking process feeling both that their ideas were valued and being comfortable sharing opinions in the context of the other ideas being voiced. They likewise emphasized the proactive, problem-solving attitude permeating the GLHS.

This practice of asking the LC about what mattered and actively listening to responses emerged during initial meetings that determined the GLHS focus. Partners first suggested focusing on avoiding long-term care for geriatric patients, but after discussions with diverse stakeholders, the focus on supporting community-dwelling older persons transitioning from hospital to home was established and the three core projects were designed. Another example was seen in how the LC recommended adding members, growing the expertise needed to inform critical analyses.

GLHS leaders sought to follow-up on explicit LC suggestions as well as assertions or beliefs. An example of the latter occurred when LC members stated that it was straightforward to schedule post-discharge primary care follow-up appointments. In response, we included questions about ease of primary care follow-up scheduling in staff interviews and included time-to-first primary care appointment as an analytic outcome. When we shared results about challenges with primary care follow-up with LC members, they listened to the findings with interest and disseminated the information to their teams for use in program evaluation and quality improvement

#### Modifying Analyses in Response to LC Input

GLHS leaders sought advice from the LC about projects and analyses. For example, although we originally planned to use 30-day readmissions as the primary outcome, we asked LC members about which transitional care measure should be used to evaluate hospitals. A lively debate led us to consider various measures including Excess Days in Acute Care (EDAC) which was subsequently included in analyses. In another example, a LC member hypothesized that some VA facilities had few VA readmissions but more community-hospital readmissions. The Data Core provided detailed analyses, confirming the LC member’s hypothesis, leading to a revision of the needs assessment interview guide.

Modified analyses in response to LC input required Data Core methodologists to be flexible, embrace the LHS iterative approach involving consideration of LC input, and foster a service mentality and willingness to collaborate. Given that no single database existed to answer questions about transitional care, the Data Core needed to assemble and transform data from diverse sources. Seeking LC input regarding analyses required time, especially when new data elements were needed to address LC suggestions. Given the complexities of observational cohort data, wealth of patient- and facility-level data, and nuances of data derived from various sources, the Data Core required a senior data scientist and experienced methodologists to respond to LC feedback. Delays in data acquisition occasionally frustrated members of the Data Core. Surmounting these challenges allowed the GLHS to tailor and improve analyses relevant to the LC.

#### Including Diverse Perspectives

The problem of transitions from hospital to home is complex and overlaps many specialties—hence the need for a multidisciplinary LC with diverse representation. Complementary expertise was necessary to provide a nuanced understanding of core issues. For example, during discussions of metrics to assess transitional care success, varying perspectives on the meaning of readmissions (whether a readmission represented a transitional “failure”) led to shifts in project outcomes. Specifically, we moved away from a single focus on 30-day risk-adjusted unplanned readmissions, to consider alternative transitional measures (e.g., EDAC) and alternative time-horizons (e.g., 7 days and 90 days post-discharge). GLHS leaders sought diversity across the following domains: services and specialties, to ensure representation from disciplines involved in the transition from hospital to home for older persons; VA versus non-VA orientation to ensure the GLHS work was broadly relevant; diverse methodological expertise to support mixed methods analyses; operational partners as well as investigators; and membership from various levels within the healthcare system including enterprise-level operational partners, regional leadership, facility and service-line leaders, and front-line clinicians. The multidisciplinary LC was unified by their shared goal of improving transitions for older persons. An unintended positive consequence of the snowballing approach to LC membership—adding members with needed experience—was including participants passionate about the target problem. During meetings, we sought to protect psychological safety and encourage participation through deliberate facilitation, thereby enhancing the opportunity for participants to share diverse and diverging opinions.

#### Navigating Organizational Complexity

Mechanisms to facilitate communication across groups were required. Members of the Administrative Core attended LC and Data Core Subcommittee meetings (for assigned subcommittees). The 18-month timeframe required GLHS leaders to attend to milestones given the ambitious portfolio of planned work. The weekly Administrative Core meetings provided a key forum for communication between cores: summarizing accomplishments, discussing challenges, and planning short-term and medium-term activities. Every other month combined Data Core meetings facilitated communication between the Qualitative and Quantitative subcommittees. The monthly newsletter provided a vehicle for communication with the entire GLHS community. We pilot-tested a meeting-minutes process, but discontinued it based on feedback from members; instead, we recorded Teams meetings for later review.

An initial communication failure was evident in the first months of the GLHS when we did not invite Data Core Quantitative Subcommittee members to LC meetings. Seeking to preserve the time of people paid to work on the Data Core, communication barriers were inadvertently created. Methodologists explained that receiving a meeting summary could not replace the value of attending LC meetings.

The time investment by LHS leaders was substantial (Table 1). LHS leadership attended the 13 regularly scheduled meetings per month (total 13.5 h); individual LHS leaders participated in 6–15 ad hoc meetings per month.

#### Serving the Healthcare System

Although the GLHS was only funded for an 18-month period, we quickly became recognized within the healthcare system as “The VA Geriatrics Learning Health System” and hence were asked by VA operational and research leadership to meet with individuals planning center proposals, research applications, and career development awards to support them, ensure that we were aware of their activities, and identify areas of collaboration. These activities were essential to system-building but were neither research nor quality improvement; they required a willingness to meet with people—often with short turn-around times—and to share ideas as well as curated datasets. For example, it was requested that we meet with the VA Hospital Medicine Analytic Team (HMAT) given their interest in examining burnout among inpatient teams. We obtained national VA employee survey data, curated those data to examine burnout among hospital medicine teams, and shared those data with HMAT.

## DISCUSSION

The LHS model is increasingly embraced globally by healthcare systems across diverse topics.^1,3,4,35,36^ This study advances the field by identifying structures and activities that LHS leaders use to support learning cycles.

An implication from the findings is that LHS must have adequate resources for leaders to engage the LC in analyses, communicate across groups, and for Data Core methodologists to have capacity for exploratory analyses. LHS leaders benefit from expertise in data science and research methodology, clinical insight regarding how care is delivered and documented, and understanding drivers of policy decision-making.

The GLHS was limited by funding for an 18-month infrastructure project. While this timeline allowed for establishing the three cores and conducting the proposed projects, it was insufficient to support additional learning cycles or follow-up on Idea Repository items. The potential harms of limited investment is that the VA enterprise might not realize long-term benefits of establishing a GLHS infrastructure, including the people involved and data assembled. The members of the cores, having acquired in-depth knowledge of the strengths and limitations of the data infrastructure, and benefiting from investments in relationship-building and communication channels, are well-placed to follow up on Idea Repository elements in further learning cycles. The future of the GLHS hinges on future funding. The ideal duration of a LHS depends on the complexity of the target problem and the activities involved in each learning cycle.^37^

The GLHS leaders intentionally adopted the stance that premature certainty about existing policies, outcomes, or improvement strategies could be detrimental to successful advancement.^17^ The focus on iterative, data-driven problem-solving conforms with Friedman’s model of integrated infrastructure supporting LHS learning cycles. A second premise fueling the GLHS infrastructure was the importance of sharing and collaboration. Although the predominant LHS model focuses on “Big Data,” a collaborative LHS emphasizes co-creation and member-focused architecture.^38^ The GLHS orientation to continuous learning relied on leadership promoting cooperation and adaptability, key LHS domains.^39^

Study limitations require description. First, although the survey suggested that the GLHS met LC member needs—an interim surrogate measure of success—assessing success can only be performed after the GLHS has been established for sufficient time so that there is potential for informing policy and improving outcomes. Second, the GLHS existed within the VA which has an exceptional data infrastructure, a culture committed to quality improvement, and a legal imperative to engage in evidence-based policy making;^18,40^ findings may not generalize to other healthcare systems. Third, the GLHS focus—transitions from hospital to home for community-dwelling older persons—was valued by VA leadership with an operational imperative to reduce adverse outcomes like readmissions; therefore, findings may not generalize to other topics addressed by LHS.

In sum, implementing a robust LHS requires active listening, responsive analyses, and including diverse perspectives. By emphasizing collaboration and iterative improvement, the GLHS fostered a supportive environment for stakeholders to engage meaningfully and inform operational and policy decision-making. Structural innovations like the Idea Repository and strategic use of a newsletter enhanced engagement and supported knowledge management. Despite the challenges of an 18-month timeline, the GLHS offers lessons about leadership strategies that can inform future LHS initiatives.

## Supplementary Information

Below is the link to the electronic supplementary material.

Supplementary Material 1 (DOCX 16.5 KB)

Supplementary Material 2 (DOC 184 KB)

## Data Availability

The data that support the findings of this study must remain on Department of Veterans Affairs servers. Please contact the corresponding author if you are interested in working with these data.
