# Supplementary material for: Leading Learning Health Systems: Strategies Used to Establish and Conduct a Learning Health System
Source: J Gen Intern Med. 2025 Sep 25;41(13):3666–75. doi: 10.1007/s11606-025-09849-0 (PMC13631195; doi:10.1007/s11606-025-09849-0)
Supplement: Supplementary file 1 [file 11606_2025_9849_MOESM1_ESM.docx]

**Geriatric LHS Learning Community Field Notes**

| Notetaker: |  |
| --- | --- |
| Date fieldnotes completed: |  |
| Date & time of event observed: |  |
| Event/Meeting (name): |  |

**Quick overview of event/interaction:**

Compose 2-3 sentences that offer a brief overview and summary of events.

**Details**: Write detailed notes on interactions, and major points of interest. Focus on non-verbal behavior, interpersonal interactions, surprises, emotions that are expressed, anything that seems to be going on “frontstage” and “backstage.”

*Welcome*

- Who participated in this Learning Community session?
- Who is entering the meeting during the meeting?
- Who was part of the QI study team?

*Reflection on pre-work*: How many people “completed” the pre-work assignment?

Who participated in the discussion regarding the summary from kickoff meeting? Were any new ideas shared? Was there any suggestions provided of someone we should contact for more information or a data source we should research?

**Summary and Comments for team discussion:**

After de-brief is completed, compose a [e.g., 3-4 sentence or more] summary that highlights key takeaway points.

In addition, please answer any of the following questions:

What is your evaluation of the meeting?

What sorts of consensus was built over the course of the meeting?

What do we learn about conducting the needs assessment or about data collection?

What was most and least surprising about the meeting?

What could be improved?
