# Supplementary material for: Leading Learning Health Systems: Strategies Used to Establish and Conduct a Learning Health System
Source: J Gen Intern Med. 2025 Sep 25;41(13):3666–75. doi: 10.1007/s11606-025-09849-0 (PMC13631195; doi:10.1007/s11606-025-09849-0)
Supplement: Supplementary file 2 [file 11606_2025_9849_MOESM2_ESM.doc]

Geriatric Learning Health Systems Learning Community
This survey aims to gather your insights on how well our Learning Community supports you and your role in supporting community-dwelling older Veterans as they transition from inpatient setting to their home. Your responses are confidential and will help shape our strategies. The survey takes about 2-5 minutes to complete and is completely voluntary. Your input is invaluable-thank you for contributing to this important work.


1.	Which of the following strategies for member	Email updates	
	engagement in the Learning Community do you find most	Office hours	
	effective?	Newsletters	
		Learning Community meetings	
	Select up to 3.	Individual meeting with evaluation team	
		Other	
	Please specify "Other"		


2.	So far, which Learning Community conversations have	 April: Role of various services in the key you found most helpful or most engaging?		elements within transitions
 May: How do you keep informed of innovative work?
Select all that apply.	 June: Metrics to evaluate transitions  August: CTraC presentation
 September: Preliminary high and low facility cohort data

Most effective	Second most effective	Third most effective
Presentation by experts
Presentation of data and its interpretation
Group discussions Collaborative problem-solving Pre-meeting materials
Other

If selected "Other" please specify.


How could Learning Community meetings be improved?	 More structured agendas
 Shorter meetings
Select all that apply.	 More opportunities for member interaction  More frequent meetings
 Clearer action items and follow-up tasks  Better meeting leadership
 Other
 None, I'm satisfied with all of the above


Please specify "Other"


To date, have you learned anything new about	Yes transitions, readmissions, geriatric populations, or	No
other topics from the Learning Community?	I don't know

If yes, what have you learned?


Have you shared information from the Learning	Yes, with others in my department or unit Community with others in the VA?	Yes, with others outside of my department or unit
Yes, with both No


The activities and information related to the Learning Community align with issues related to improving patient transitions from inpatient to home.
Not at all
accurate

Slightly accurate	Moderately
accurate

Mostly accurate	Completely
accurate


This Learning Community is more appealing/useful to me	Agree than other similar professional communities in which I	Disagree am a member?

Please share why you agree or disagree.
